# Assessment of Disparities in COVID-19 Testing and Infection Across Language Groups in Seattle, Washington

**DOI:** 10.1001/jamanetworkopen.2020.21213

**Published:** 2020-09-24

**Authors:** H. Nina Kim, Kristine F. Lan, Esi Nkyekyer, Santiago Neme, Martine Pierre-Louis, Lisa Chew, Herbert C. Duber

**Affiliations:** 1 Department of Medicine, University of Washington School of Medicine, Seattle; 2 Division of Allergy and Infectious Disease, University of Washington School of Medicine, Seattle; 3 Harborview Medical Center, Seattle, Washington; 4 Department of Emergency Medicine, University of Washington, Seattle; 5 Institute for Health Metrics and Evaluation, University of Washington, Seattle

## Abstract

This cross-sectional study evaluates the proportion of patients tested for coronavirus disease 2019 (COVID-19) and the proportion of positive cases, using language as a surrogate for immigrant status.

## Introduction

Clinicians from New York, New York, have raised the alarm that the coronavirus disease 2019 (COVID-19) pandemic has taken a disproportionate toll on their local immigrant communities.^1^ Immigrants may be more susceptible to exposure because more of them work in essential industries or reside in larger multigenerational households.^2^ Limited English language proficiency (LEP) or low health literacy can present challenges to effective communication about disease transmission.^3^ Worries about stigma, deportation, or livelihood may supersede those of a health threat, however serious.^4^ It remains unclear whether these disparities have resulted in lower comparative access to testing for severe acute respiratory syndrome coronavirus 2 (SARS-CoV-2), a patient-initiated option in all but congregant settings, or in higher rates of infection among immigrants. To clarify this issue, we evaluated the proportion of patients who completed testing and the proportion of positive cases using language as a surrogate for immigrant status.

## Methods

This analysis was approved by the University of Washington human subjects division. A waiver of consent was granted because the study is a retrospective data analysis of deidentified data and not considered human subjects research. This cross-sectional study follows the Strengthening the Reporting of Observational Studies in Epidemiology (STROBE) reporting guideline.

The University of Washington Medicine system comprises 3 hospitals and more than 300 clinics across the Puget Sound region. Nasopharyngeal samples were tested for SARS-CoV-2 using a real-time reverse transcriptase–polymerase chain reaction test developed by the University of Washington Virology laboratory.^5^ After identifying all living patients who had at least 1 encounter (including telephone or telemedicine) in our system from January 1, 2019, to February 28, 2020, we assessed SARS-CoV-2 testing from February 29 when testing first started to May 31, 2020, as (1) the proportion of those tested and (2) the proportion of those who tested positive over those tested, and calculated 95% CIs using a standard error of the proportion formula. Results were stratified by language, designated by the patient and captured on registration as their preferred spoken language. Data analysis was performed using R statistical software version 3.6.3 (R Project for Statistical Computing) on August 4, 2020.

## Results

**Figure 1.**
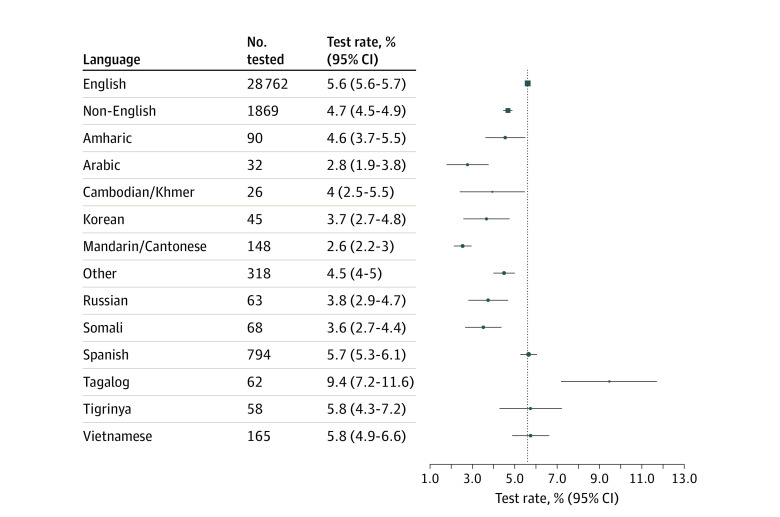
Proportion of Patients Tested for Severe Acute Respiratory Syndrome Coronavirus 2 (SARS-CoV-2) by Language Estimates and 95% CIs are shown for English, pooled non-English languages, and the 12 most populous non-English languages. Language was missing for 10 844 (1.9%) of 562 242 individuals assessed.

**Figure 2.**
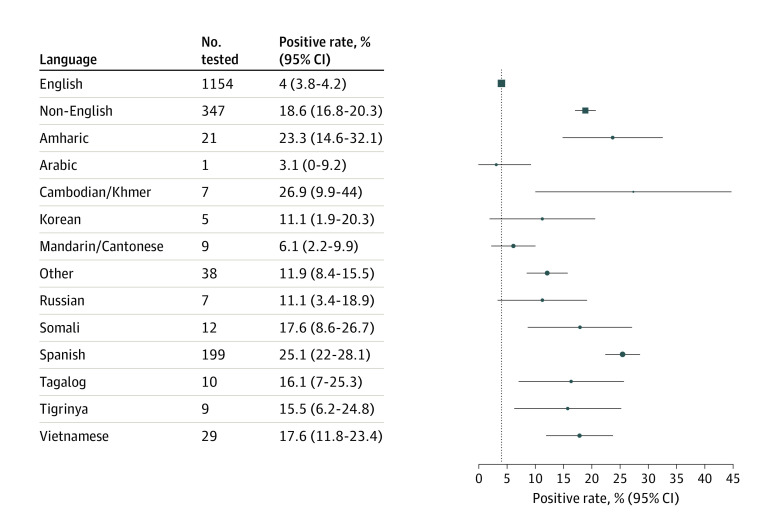
Proportion of Patients Testing Positive for Severe Acute Respiratory Syndrome Coronavirus 2 (SARS-CoV-2) by Language Estimates and 95% CIs are shown for English, pooled non-English languages, and the 12 most populous non-English languages. Language was missing for 291 (1%) of 30 925 individuals tested.

Overall, 30 925 of 562 242 patients (5.5%) underwent SARS-CoV-2 testing. The mean (SD) age of the patients was 49 (18.3) years, and 16 152 (52.2%) were women. Of these, 1869 (6.0%) were non-English speakers. Non-English speakers were overall less likely to have completed testing compared with English-speakers (4.7% [95% CI, 4.5%-4.9%] vs 5.6% [95% CI, 5.6%-5.7%]) but the proportion tested varied across language groups (Figure 1). Notably, the proportion of positive cases was 4.6-fold higher among non-English speakers overall (18.6%; 95% CI, 16.8%-20.4%) compared with English speakers (4.0%; 95% CI, 3.8%-4.2%) (Figure 2). This excess risk was observed across multiple languages and in the 3 largest non–English-speaking groups—Spanish, Vietnamese, and Amharic—reflecting the larger immigrant communities in King County, WA.

## Discussion

A deeper understanding of disparities in health care requires a closer look at structural inequities and other social determinants of health. In the US, LEP has been shown to be associated with quality of care, health care–seeking behavior, and health outcomes.^6^ Our study found that despite the availability of interpreter services across clinical locations, non–English-speaking patients in our health system were tested less frequently for COVID-19 and had significantly higher burden of infection.

Interestingly, we found substantial heterogeneity in both test and test positivity rates by language. Higher testing rates were noted among those language groups living in neighborhoods where community-based testing strategies were implemented. Our outreach efforts, which included mobile clinics and drive-up testing, was implemented in partnership with local health coalitions representing communities of color to overcome testing barriers and should be considered where limited access and disproportionate rates of infection are a concern.

Our descriptive analysis of the distribution of COVID-19 testing and infection among LEP communities within a single health system should be interpreted within the context of certain limitations. Testing rates shown here may not reflect the true testing rate of the LEP community in our region because we examined only University of Washington Medicine testing and defined our patient population by requiring documented contact in the past year, which might have introduced selection bias. Second, language may not accurately represent how immigrant community members identify. Third, we did not adjust for potential confounding or mediating factors to understand the true association between LEP and COVID-19 testing and infection. Until we routinely examine quality of care outcomes by granular demographic measures, such as language or country of origin, disparities in care for immigrants can continue to remain hidden.
